# Molecular interplays of the *Entamoeba histolytica* endosomal sorting complexes required for transport during phagocytosis

**DOI:** 10.3389/fcimb.2022.855797

**Published:** 2022-10-27

**Authors:** Cecilia Bañuelos, Abigail Betanzos, Rosario Javier-Reyna, Ausencio Galindo, Esther Orozco

**Affiliations:** ^1^ Coordinación General de Programas de Posgrado Multidisciplinarios, Programa de Doctorado Transdisciplinario en Desarrollo Científico y Tecnológico para la Sociedad, Centro de Investigación y de Estudios Avanzados (CINVESTAV), Mexico City, Mexico; ^2^ Investigadores por Mexico, Consejo Nacional de Ciencia y Tecnología (CONACYT), Mexico City, Mexico; ^3^ Departamento de Infectómica y Patogénesis Molecular, Centro de Investigación y de Estudios Avanzados (CINVESTAV), Mexico City, Mexico

**Keywords:** cargo sorting, EhRabs, EhADH, endosome maturation, late endosome/MVB, intraluminal vesicles, LBPA, Vps

## Abstract

*Entamoeba histolytica*, the causative agent of human amoebiasis, exhibits a continuous membrane remodelling to exert its virulence properties. During this dynamic process, the Endosomal Sorting Complexes Required for Transport (ESCRT) machinery is a key player, particularly in phagocytosis, a virulence hallmark of this parasite. In addition to ESCRT, other molecules contribute to membrane remodelling, including the EhADH adhesin, EhRabs, actin, and the lysobisphosphatidic acid (LBPA). The endocytosis of a prey or molecules induces membrane invaginations, resulting in endosome and multivesicular bodies (MVBs) formation for cargo delivery into lysosomes. Alternatively, some proteins are recycled or secreted. Most of these pathways have been broadly characterized in other biological systems, but poorly described in protozoan parasites. Here, we encompass 10 years of ESCRT research in *E. histolytica*, highlighting the role of the ESCRT-I and ESCRT-III components and the EhADH and EhVps4-ATPase accessory proteins during phagocytosis. In particular, EhADH exhibits a multifunctional role along the endocytic pathway, from cargo recognition to endosome maturation and lysosomal degradation. Interestingly, the interaction of EhADH with EhVps32 seems to shape a concurrent route to the conventional one for MVBs biogenesis, that could optimize their formation. Furthermore, this adhesin is secreted, but its role in this event remains under study. Other components from the endosomal pathway, such as EhVps23 and LBPA, are also secreted. A proteomic approach performed here, using an anti-LBPA antibody, revealed that some proteins related to membrane trafficking, cellular transport, cytoskeleton dynamics, and transcriptional and translational functions are secreted and associated to LBPA. Altogether, the accumulated knowledge around the ESCRT machinery in *E. histolytica*, points it out as a dynamic platform facilitating the interaction of molecules participating in different cellular events. Seen as an integrated system, ESCRTs lead to a better understanding of *E. histolytica* phagocytosis.

## Introduction

The need for cell nourishment directs life activities from microorganisms to humans. To allow the uptake of nutrients, numerous molecules interact to perform diverse functions that involve distinct cell organelles. The plasma membrane (PM) acts as the interphase with the environment, where nutrients are present, and connects with the membranous system in the cytoplasm, where the ingested material is processed (Simpson, 2020). The nutrients uptake first depends on the interaction and turnover of receptors and ligands. Proteins forming channels and vesicles, and carrier proteins, act as vehicles to transport the prey (target cells) or cargo (molecules), or to biochemically transform it. The presence and quantity of factors involved in all these events are finely regulated in time and cellular location. Once ingested, the nutrients are degraded into simple compounds and, on the other hand, the cell recovers useful molecules for further utilization. Molecular cross-talking and membrane remodelling maintain the balance in all these processes and are the core of these events (Arlt et al., 2015; Palm and Thompson, 2017).

*Entamoeba histolytica*, the parasite causative of human amoebiasis, is a professional devourer of bacteria and eukaryotic cells (Orozco et al., 1983). During capture, ingestion and digestion of the prey, the PM and internal membranes exhibit a particular dynamic activity, that, in addition to the movement of the prey inside the cell, transport the proteins that direct the events. When trophozoites invade human tissue, the nutrients are captured by endocytosis, phagocytosis and trogocytosis (Labruyère and Guillén, 2006; Nozaki and Nakada-Tsukui, 2006; Ralston et al., 2014).

In response to an infection, both, the innate and adaptive immune responses are activated to control and eliminate invasive *E. histolytica.* Infiltrating trophozoites are attacked by cells of the complement system, and molecules present in the blood. Parasites are recognized by dendritic cells, which then activate CD4+ and CD8+ T cells, for developing a cellular immune response. CD4+ T cells produce IFN-γ, IL-4, and IL-5, whereas CD8+ T cells produce IL-17. IL-17 enhances secretion of IgA antibodies (humoral immune response) into the colonic lumen. IFN-γ stimulates macrophages to produce nitric oxide (NO) and neutrophils to release extracellular traps (NET). NO can directly kill amoebas, whereas NET can trap and immobilize them (Ávila et al., 2016; Díaz-Godínez et al., 2018; Uribe-Querol and Rosales, 2020).

In turn, trophozoites use multiple pathogenic factors to resist the immune response and continue its survival and pathogenesis (Nakada-Tsukui and Nozaki, 2016). In the lumen of the large intestine, glycosidases and proteinases secreted from trophozoites, are involved in the degradation of the mucin mucus layer. The cysteine proteinase (EhCP)-A5 binds to and activates integrins on endothelial cells, leading to NLRP3 inflammasome formation. Also, the galactose and N-acetylgalactosamine (Gal/GalNAc) lectin binds to the Toll-like receptor 2, leading to NF-κB activation and release of inflammatory cytokines (IL-1, IL-6, IL-8, IL-12, IFN-γ and TNF-α) (Nakada-Tsukui and Nozaki, 2016; Uribe-Querol and Rosales, 2020).

The EhCPADH complex, integrated by the EhADH adhesin and the EhCP112 cysteine protease (García-Rivera et al., 1999), and the prostaglandin E2, secreted from the amoeba, disrupt tight junctions of epithelial cells (Lejeune et al., 2011; Betanzos et al., 2013). Phagocytosis and trogocytosis are also involved in the removal of epithelial cells and invasion into the tissue (Labruyère and Guillén, 2006; Ralston et al., 2014).

By their medical importance, their high activity during host invasion and the unique atypical organelles that the trophozoites have (Nozaki and Nakada-Tsukui, 2006; Smith and Guillen, 2010; Betanzos et al., 2019), they constitute an excellent system to study membrane remodelling, vesicular trafficking and their participation in the target cell attack.

Our laboratory has studied molecules involved in phagocytosis. Data have shown that the Endosomal Sorting Complexes Required for Transport (ESCRT) machinery is involved in cellular functions, including phagocytosis, that require vesicle formation and membrane scission and repair. The ESCRT proteins interact with other molecules and together, they construct a chain of events that maintain the continuity of the process. Each one of the events and molecules of this chain are important, and if one of them is affected, the entire process is disturbed (López-Reyes et al., 2011; Avalos-Padilla et al., 2018; Galindo et al., 2022).

In eukaryotes, the ESCRT machinery is formed by the complexes: ESCRT-0 (Vps27/Hrs, Hse1/STAM1), ESCRT-I (Vps23/Tsg101, Vps28, Vps37, Mvb12), ESCRT-II (Vps22/EAP30, Vps25/EAP20, Vps36/EAP45) and ESCRT-III (Vps20/CHMP6, Vps32/CHMP4, Vps24/CHMP3, Vps2/CHMP2) and the ESCRT accessory proteins (Bro1/Alix, Vps4-ATPase and Vta1/LIP5) (in parenthesis the names of the *Saccharomyces cerevisiae*/*Homo sapiens* proteins of each complex are indicated) (**Table 1**) (Hurley, 2010; Schuh and Audhya, 2014). The majority of the genes and proteins of the ESCRT machinery are present in *E. histolytica* (**Table 1**) (López-Reyes et al., 2011). ESCRT proteins change their cell location through phagocytosis, according to the advance of the event (López-Reyes et al., 2010). Their knock down or overexpression have repercussions in the rate of phagocytosis and in the *in vitro* and *in vivo* virulence expression (López-Reyes et al., 2010; Bañuelos et al., 2012; Avalos-Padilla et al., 2015; Avalos-Padilla et al., 2018; Galindo et al., 2021; Galindo et al., 2022), highlighting their role in membrane transformation and in vesicles, tubes and tunnel-like structures formation, which in turn contribute to the transport of cargo, or the prey.

**Table 1:** Members of the ESCRT machinery in *E. histolytica, H. sapiens* and *S. cerevisiae*.

Complex	*E. histolytica*	Domains	*S. cerevisiae*	Domains	*H. sapiens*	Domains
Ancestral ESCRT-0	EhTom1	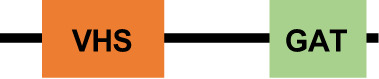	——		Tom1 TomL1 TomL2	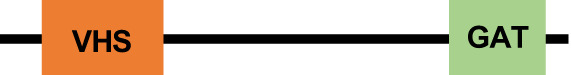
ESCRT-0	EhVps27	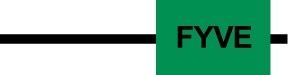	Vps27	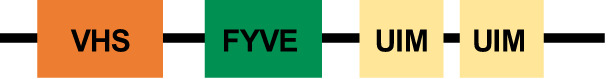	Hrs	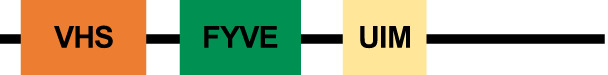
	EhHse1	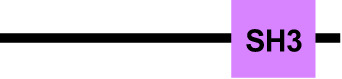	Hse1	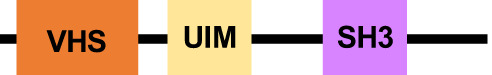	STAM1 STAM2	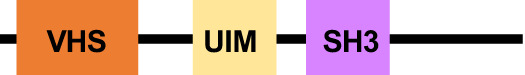
ESCRT-I	EhVps23	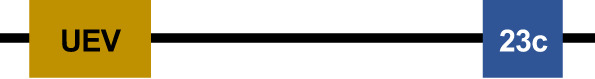	Vps23	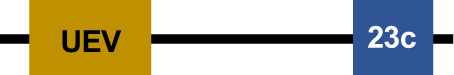	Tsg101	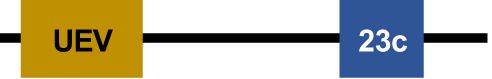
	EhVps28	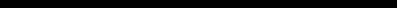	Vps28	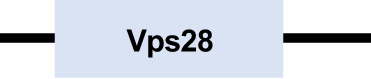	Vps28	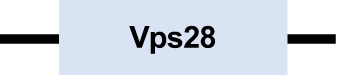
	EhVps37D	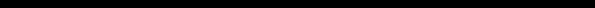	Vps37	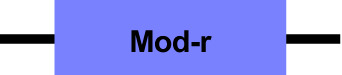	Vps37A Vps37B Vps37C Vps37D	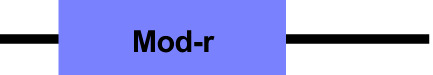
	——		Mvb12	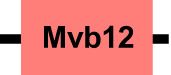	Mvb12A Mvb12B	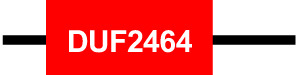
ESCRT-II	EhVps22	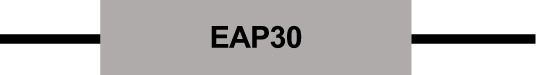	Vps22	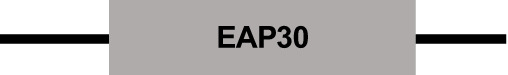	EAP30	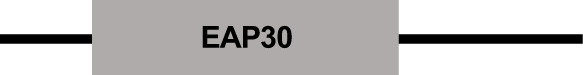
	EhVps25	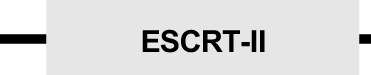	Vps25	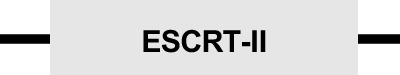	EAP20	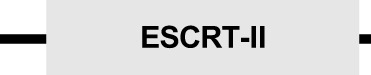
	EhVps36	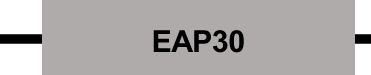	Vps36	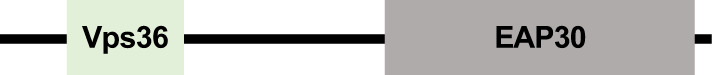	EAP45	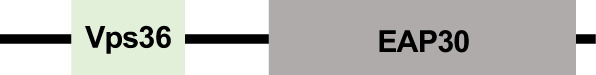
ESCRT-III	EhVps2	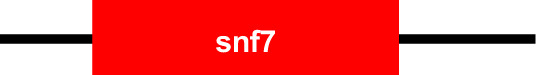	Vps2	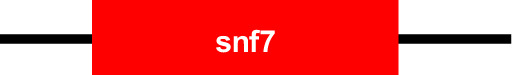	CHMP2A CHMP2B	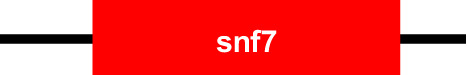
	EhVps20	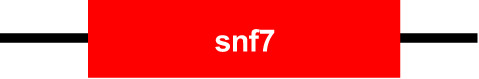	Vps20	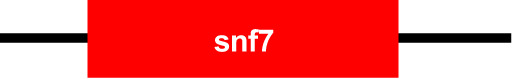	CHMP6	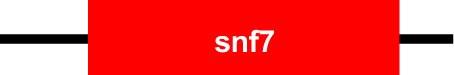
	EhVps24	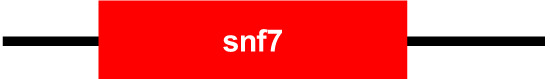	Vps24	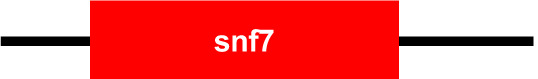	CHMP3	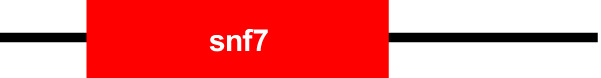
	EhVps32	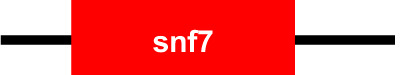	Vps32	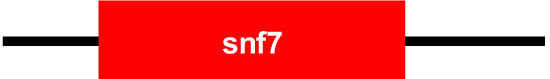	CHMP4A CHMP4B CHMP4C	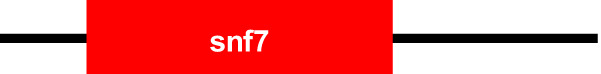
Accessory	EhVps4	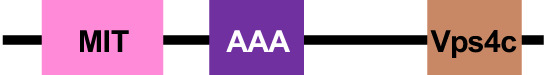	Vps4	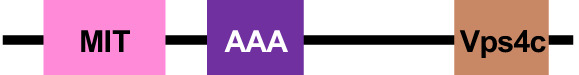	Vps4 A Vps4 B	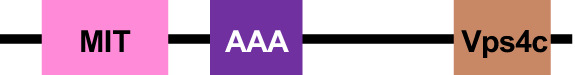
	EhADH	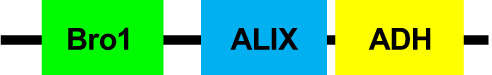	Bro1	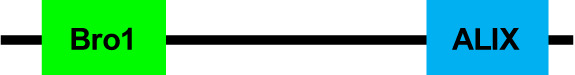	Alix HDPTP	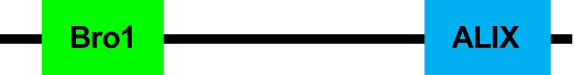
	EhVta1	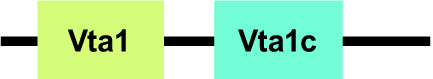	Vta1	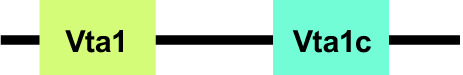	LIP5	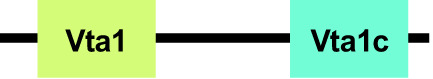
	EhDoa4	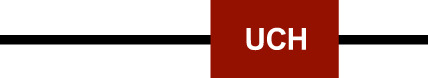	Doa4	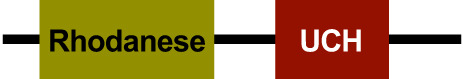	UBPY	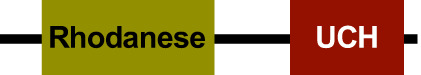

To perform phagocytosis, the trophozoites need first to be attracted to and make contact with the prey (Orozco et al., 1985). Several ESCRT proteins associate to EhADH and Gal/GalNAc (Bañuelos et al., 2012; Avalos-Padilla et al., 2015; Avalos-Padilla et al., 2018; Galindo et al., 2021), both involved in the adherence of trophozoites to the target cells (García-Rivera et al., 1999; Lopez-Vancell et al., 2000). Cellular signals initiate the pseudopodia formation and the capture of the prey. In this step, ESCRT proteins interact with actin, RabB and possibly other Rab proteins (Javier-Reyna et al., 2019). In general, Rab small GTPases play a fundamental role in signalling and activation of distinct molecules involved in the process (Saito-Nakano et al., 2005; Verma et al., 2020). ESCRT proteins have also been detected in phagosomes, late endosomes (LE), multivesicular bodies (MVBs) and their intraluminal vesicles (ILVs), in secreted vesicles, and in the MVBs fused to lysosomes (López-Reyes et al., 2010; Bañuelos et al., 2012; Avalos-Padilla et al., 2015; Avalos-Padilla et al., 2018; Galindo et al., 2021; Galindo et al., 2022). These sequential events allow first, the transport of the prey through compartments containing different enzymes and molecules, ending in the prey digestion for recycling useful proteins, or in the secretion of others. EhADH acts as an adhesin and then, through the whole process, as a scaffold protein carrying molecules and interacting with distinct ESCRT proteins (Bañuelos et al., 2012; Avalos-Padilla et al., 2015; Avalos-Padilla et al., 2018; Galindo et al., 2021; Galindo et al., 2022). Given the importance of endocytosis, particularly phagocytosis, and movement, in the *E. histolytica* virulence, we reviewed here the main known facts on membrane remodelling and protein transport during phagocytosis, with special emphasis in the role of the ESCRT machinery.

## Participation of ESCRT in recognition and capturing of the prey and cargo

### Target recognition

To perform the capturing of the prey, in general, trophozoites require to adhere to the target. The virulent strains of *E*. *histolytica* display a high rate of pinocytosis (micropinocytosis and macropinocytosis) and endocytosis (trogocytosis and phagocytosis) (Rodríguez and Orozco, 1986; Laughlin et al., 2004; Bettadapur and Ralston, 2020). Pinocytosis is the process by which the trophozoites absorb extracellular fluids and compounds (Laughlin et al., 2004), while in endocytosis, the cells capture macromolecules, cell surface components and even whole cells that include red blood cells (RBCs), live and apoptotic mammalian cells, and bacteria (Labruyère and Guillén, 2006). So far, there are no reports precising the mechanisms used by *E. histolytica* to discriminate among particles, molecules or whole cells to be internalized.

Endocytic processes include phagocytosis, that refers to live whole, damaged or dead cells engulfment; and trogocytosis, where trophozoites ingest part of the living cells (Ralston et al., 2014; Bettadapur and Ralston, 2020; Nakada-Tsukui and Nozaki, 2021). The PM invagination and the formation of vesicles and vacuoles required for these processes, imply the renewal of the PM every 30 min (Doherty and McMahon, 2009).

**Figure 1:**
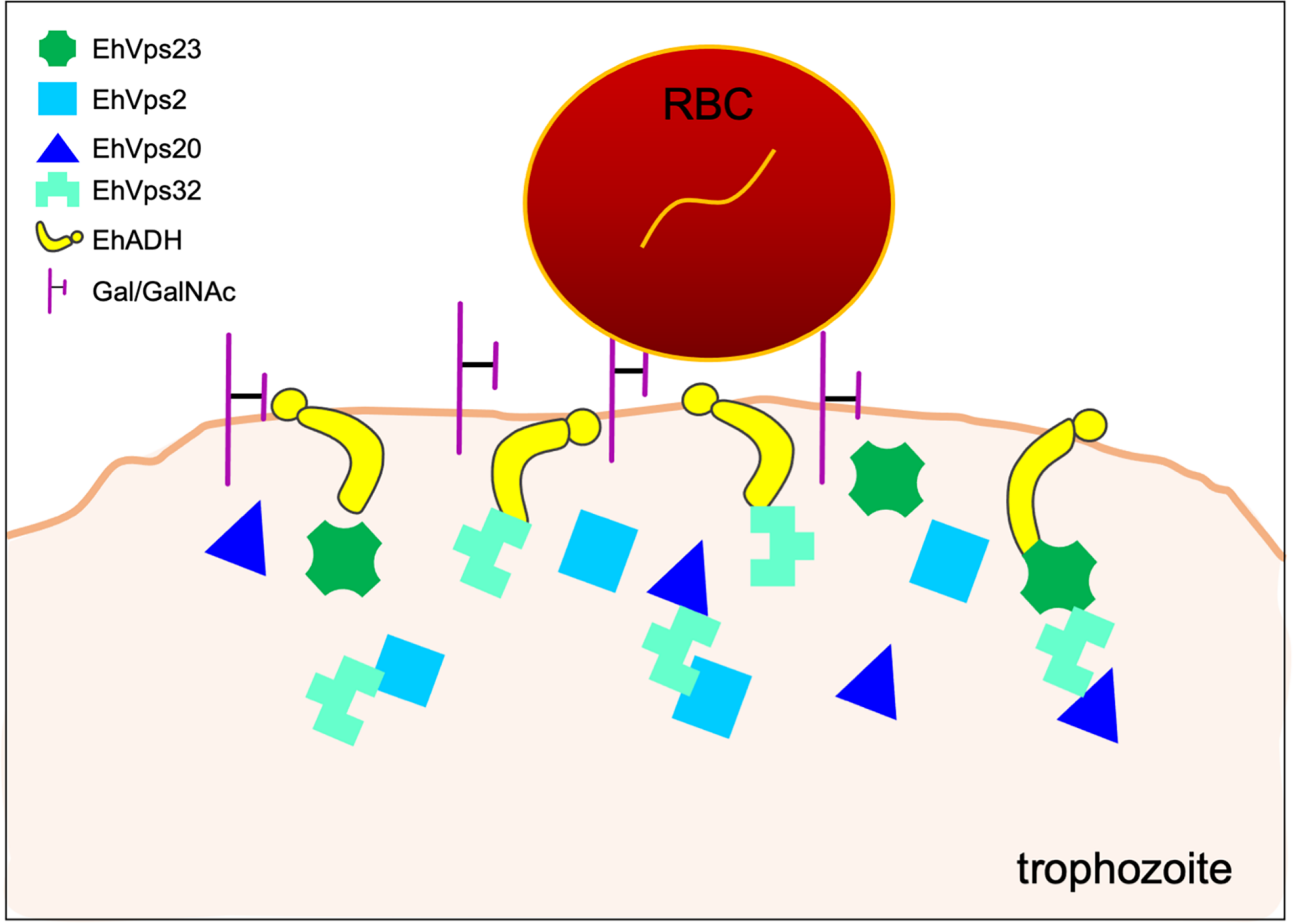
Initial contact of trophozoites with RBCs. During the capture of the prey by trophozoites, the EhADH adhesin and the Gal/GalNAc lectin participate. At the contact site, some ESCRT-I and ESCRT-III components are localized, suggesting their role in this event.

Particularly, phagocytosis begins with the adherence of trophozoites to target cells (Christy and Petri, 2011). The most characterized molecules in the primary contact are the Gal/GalNAc lectin (Singh et al., 2016) and the EhADH adhesin (**Figure 1**) (García-Rivera et al., 1999). Other proteins involved in the contact to RBCs and human colorectal adenocarcinoma (Caco-2) cells, include the C2-domain–containing protein kinase (EhC2PK) (Babuta et al., 2020) and the lysine and glutamic acid rich protein (KERP1) (Seigneur et al., 2005), respectively.

In the first step of phagocytosis, EhVps2, EhVps20, and EhVps32 proteins from the ESCRT-III complex (Avalos-Padilla et al., 2015; Avalos-Padilla et al., 2018) and EhVps23 from the ESCRT-I complex (Galindo et al., 2021) have been localized at PM, just in the place where the PM of trophozoites makes contact with the membrane of the target cell. During erythrophagocytosis, EhVps32 appears associated to the Gal/GalNAc lectin (Avalos-Padilla et al., 2015), while several proteins of ESCRT-I and ESCRT-III interact with the EhADH adhesin (Bañuelos et al., 2012; Avalos-Padilla et al., 2015; Avalos-Padilla et al., 2018; Galindo et al., 2021) (**Figure 1**). Future research will elucidate whether these ESCRT proteins help in some way to the adherence function.

The Gal/GalNAc lectin is a heterodimer that comprises a transmembrane heavy subunit (Hgl, 170 kDa) and a glycosylphosphatidylinositol (GPI)-anchored light subunit (Lgl, 35/31 kDa), glycoproteins linked by disulphide bonds. The Hgl subunit contains a carbohydrate recognition domain that binds to D-galactose and N-acetyl-D-galactosamine and it is important for cell adhesion and extracellular matrix (ECM) degradation, and intervenes in both, colonization and contact-dependent cytotoxicity. Recent studies have suggested a role of Hgl in the ECM-mediated actin dot formation (Petri et al., 2002).

On the other hand, EhADH is a multifunctional protein that contains at its N-terminal a Bro-1 domain, which makes it a member of the ALIX family (Bañuelos et al., 2005; Montaño et al., 2017). Its ability of binding to the target cell is conferred by an adherence domain present at the C-terminal (Arroyo and Orozco, 1987; García-Rivera et al., 1999). Monoclonal antibodies against the adherence domain inhibit trophozoite adhesion to and phagocytosis of erythrocytes (García-Rivera et al., 1999). In epithelial cells, this adhesin binds to proteins of the intercellular junctions, such as tight and adherens junctions and desmosomes, contributing to the epithelial damage and invasion (Betanzos et al., 2013; Hernández-Nava et al., 2017; Betanzos et al., 2018). These findings support the former evidences about the presence of trophozoites tightly adhered to the intercellular space between two epithelial cells, where these junctions are located (Martinez-Palomo et al., 1985).

Of note, DNA vaccination of animals with the *Ehadh* and *Ehcp112* genes, improves the immune response of hamsters against *E. histolytica*, and protects the animals from the damage caused in the liver by virulent amoeba strains (Martínez et al., 2009). In addition, mutant trophozoites with the *Ehadh* gene silenced show a reduction (30%) in their rate of erythrophagocytosis (Ocádiz-Ruiz et al., 2016). In contrast, EhADH overexpression increases (76%) the rate of phagocytosis by trophozoites (Bañuelos et al., 2012).

EhADH is also an important accessory protein of the ESCRT machinery, and it frequently appears associated to other ESCRT members (Bañuelos et al., 2012; Avalos-Padilla et al., 2015; Avalos-Padilla et al., 2018; Galindo et al., 2021). It binds to EhVps32 and EhVps23 through its Bro1 domain (Bañuelos et al., 2012; Galindo et al., 2021).

Furthermore, the EhCPADH complex, the Gal/GalNAc lectin, and the EhVps32 (ESCRT-III) protein co-localize at the site of contact of trophozoites with RBCs (Avalos-Padilla et al., 2015). In this same attachment place, EhADH appears together with the EhVps23 (ESCRT-I) (Galindo et al., 2021), highlighting the role of the ESCRT machinery accompanying the Gal/GalNAc lectin and EhADH protein in their function as receptors. At the site of contact with RBCs, EhVps2 and EhVps24 are also present, suggesting that these ESCRT components also bind to surface proteins (Avalos-Padilla et al., 2018). Overall, these data indicate that several molecules participate to allow the trophozoites the specific contact with their prey.

EhADH also associates to the cholesterol-trafficking proteins EhNPC1 and EhNPC2, suggesting an extra role for this molecule in the uptake and transport of this essential lipid toward cellular membranes (Bolaños et al., 2016). Although no relationship of cholesterol and the ESCRT machinery has been described yet, it is well known that cholesterol improves the adherence of trophozoites to the host cells and to the ECM (Mittal et al., 2008). On the other hand, several ESCRT proteins possess lipid-binding domains (Teo et al., 2006); thus, it is possible that these molecules act and interact with cholesterol during the membranes remodelling in phagocytosis.

Membranes of trophozoites are mainly composed by phospholipids and cholesterol (Das et al., 2002). Particularly, the vesicle’s lipid and protein composition varies according to the function of the vesicle and its content (Goldston et al., 2012b; Castellanos-Castro et al., 2020), as shown using the model of giant unilamellar vesicles (GUVs) for reconstructing the ESCRT-III subunits assembly (Avalos-Padilla et al., 2018). To mimic the endosomal membranes composition, authors probed several lipids combinations, resulting phosphocholine:phosphoserine:cholesterol: phosphatidylinositol 3, phosphate (PI3P) in a 62:10:25:3 ratio, the optimal one (Avalos-Padilla et al., 2018).

In addition to the transport of molecules during the vesicular trafficking, lipids also participate in the dynamic membrane fusion and fission. Several lipids have already been detected in the trophozoites during the capture and ingestion of the prey (Mittal et al., 2008; Welter et al., 2011; Castellanos-Castro et al., 2020). Phosphatidylinositol, a member of the family of glycerophospholipids, is phosphorylated at all combinations of D-3, 4, and 5 positions of the inositol ring, forming seven isotypes of phosphatidylinositol phosphates (PIPs). In eukaryotes, PIPs are localized at the PM and in membrane regions connecting them to the cytoskeleton. For instance, PIP2 is a critical regulator of actin polymerization and cytoskeleton/membrane linkages. The binding of cytoskeletal proteins to membrane PIP2 might alter lateral or transverse movement of lipids to affect raft formation or lipid asymmetry (Zhang et al., 2012). In *E. histolytica*, this lipid family is involved in phagocytosis and trogocytosis (Goldston et al., 2012a; Watanabe et al., 2020). PI(4,5)P2 is localized in the PM and mediates the signalling during cell adhesion (Goldston et al., 2012a). Nevertheless, it has not been experimentally probed whether the trophozoites lipids interact with the ESCRT machinery proteins since the first contact, as in other eukaryotes (Teo et al., 2006).

The *E. histolytica* lipophosphoglycan (LPPG), mainly found in virulent strains, has been proposed as a molecule involved in the contact of the trophozoites and target cells (Stanley et al., 1992; Moody et al., 1998).

However, so far, it has not been reported the interaction between the LPPG and subunits of the ESCRT machinery, or with other proteins involved in phagocytosis.

### Phagocytic cup formation

In eukaryotes, the receptor-ligand clustering produces changes in the PM composition and topology, triggering activation pathways (Zhdanov, 2017). In *E. histolytica*, the receptor-ligand clustering leads to the activation of the cytoskeleton, forming pseudopodia that surround the target, developing the phagocytic cup and participating in the active movement of the trophozoites (Verma and Datta, 2017). During the phagocytic cup formation, Ca^2+^ signalling (Jain et al., 2008) plays an important role in the recruitment of actin (Mansuri et al., 2014). There is a group of calcium-binding proteins (CaBPs), such as EhCaBP1 and EhCaBP3 known to regulate the dynamics of the cytoskeleton (Bhattacharya et al., 2006). These proteins interact with actin and phosphatidylserine that, along with actin remodelling and other molecules, produce the membrane deformation to form the phagocytic cup (Jain et al., 2008; Aslam et al., 2012).

**Figure 2:**
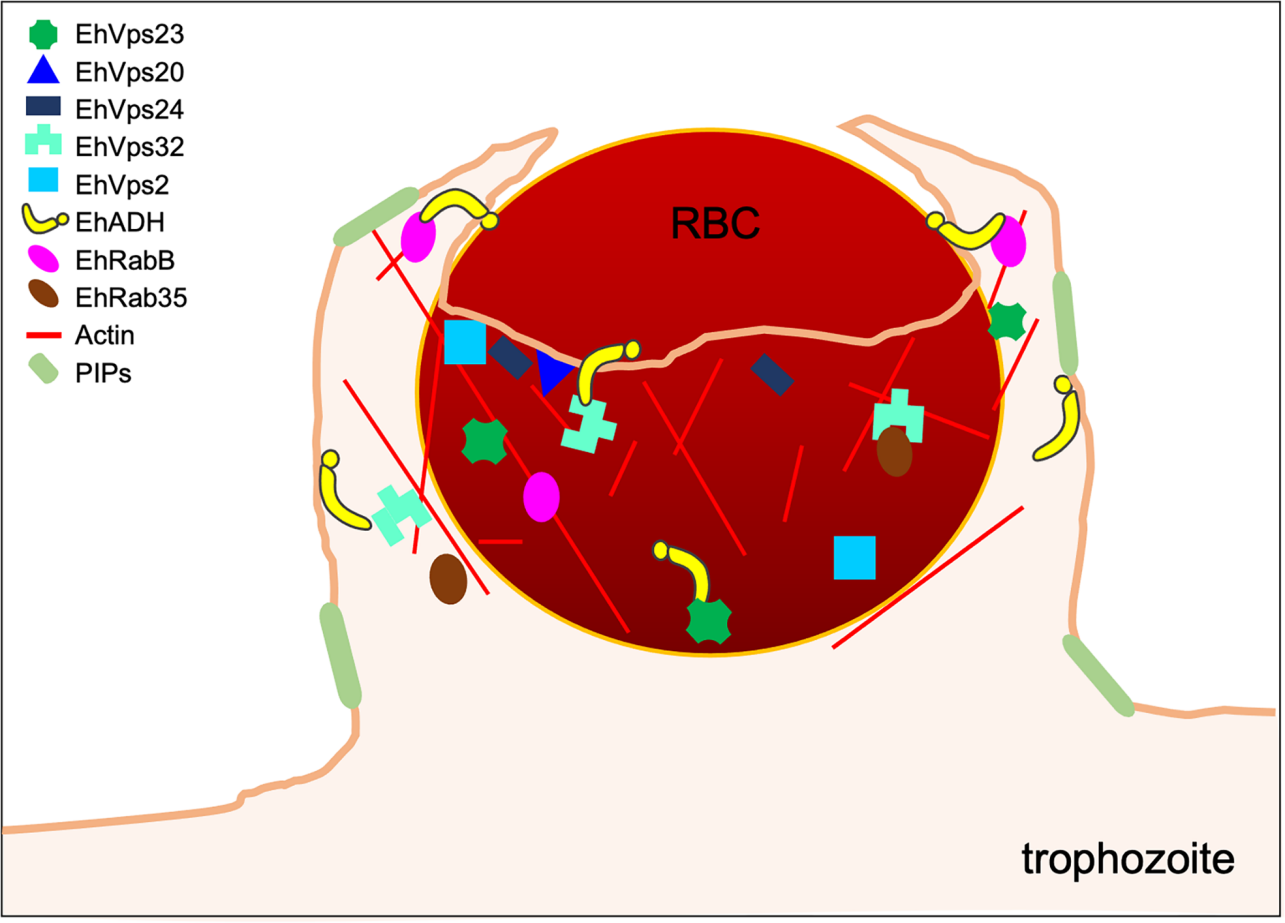
Phagocytic cup formation. During RBCs internalization, the PM deformation involves the actin cytoskeleton and some small GTPases, as EhRab35, to generate the phagocytic cup. Other molecules have been localized in these structures, such as lipids (PIPs), EhRabB, and ESCRT-I, ESCRT-III and ESCRT accessory proteins.

Other proteins involved in the organization and regulation of actin cytoskeleton during the cup formation are the small GTPases, such as the Rho, Rab, and Arf families (Bosch and Siderovski, 2013). EhRab21 and its effector EhC2B, which binds to phosphatidylserine in the presence of calcium, are localized in the advancing tips of the phagocytic cup, where EhC2B catalyses actin polymerization (Tripathi et al., 2020). EhRab35 is also present in the phagocytic cups (**Figure 2**). In addition, the expression of an EhRab35 dominant negative in trophozoites, reduces the formation of phagocytic cups (Verma and Datta, 2017). By *in silico* analysis, it has been demonstrated that EhRabB binds to the EhADH Bro1 domain through its switch I zone; and to actin by the switch I and II regions (Javier-Reyna et al., 2019). These regions are crucial for the switching between GTP- and GDP-bound forms (Bos et al., 2007), facilitated by a guanine nucleotide exchange factor (Lee et al., 2009). In human, these states exhibit structural differences, allowing selective recognition of Rabs by regulatory and effectors proteins in a nucleotide-dependent manner (Pylypenko et al., 2018). In *E. histolytica*, by immunoprecipitation and immunofluorescence assays, it has been demonstrated that EhRabB binds to EhADH and actin, and all of them co-localize at the phagocytic cup (**Figure 2**) (Javier-Reyna et al., 2019). In addition, EhVps23 from the ESCRT-I complex, and EhVp32, a member of ESCRT-III, are also localized at the phagocytic cups (**Figure 2**) (Avalos-Padilla et al., 2015; Galindo et al., 2021). Thus, it is plausible to hypothesize that these proteins are directly or indirectly interacting with EhRabB and actin.

The lipids such as PI3P and PI(3,4,5)P3 are localized at the phagocytic (**Figure 2**) and trogocytic cups, where PI3P-binding proteins are recruited though FYVE domains (Powell et al., 2006; Nakada-Tsukui et al., 2009; Byekova et al., 2010). The *E. histolytica* genome is predicted to encode eleven FYVE domain-containing proteins (FPs) (Nakada-Tsukui et al., 2009). In yeast and human, the FYVE is a characteristic domain of the Vps27/HRS protein from the ESCRT-0 complex (Katzmann et al., 2003). In *E. histolytica*, the EhVps27 protein also harbors the FYVE domain (López-Reyes et al., 2011). Another FP protein, is EhFP4, which interacts with PIPs on the PM, thereby recruiting proteins such as EhRacC and EhRacD, both involved in the polymerization of actin filaments at the phagocytic cup (Nakada-Tsukui et al., 2009; Watanabe et al., 2020).

These findings, point out to the participation of some lipids, small GTPases and its effectors, together with the actin cytoskeleton and some ESCRT members during the prey capture, including the phagocytic cup formation and the actin cytoskeleton organization.

## The ESCRT machinery and endosomal compartments during phagocytosis

### Early and late endosomes

Phagocytosis by professional phagocytes such as macrophages and immature dendritic cells in humans, and of course *E. histolytica* trophozoites, involves prey recognition, capture, and internalization by fusion of the extended pseudopodia. Once the prey has been internalized, it passes through a series of pleomorphic tubule-vesicular compartments, collectively called endosomes (Schuh and Audhya, 2014). In human and yeast, endosomes undergo maturation from EE to LE, which involves decreased luminal pH (6.0-4.9), a change in PIPs composition, and recruitment and activation of Rabs (Scott et al., 2014). In *E. histolytica*, the difference between EE and LE is not entirely clear, since the composition of PIPs (PI3P, PI[4,5]P2 and PI[3,4,5]P3) is similar in distinct stages of the endosome maturation, and there are no conclusive specific markers for each type of endosomes.

In trophozoites, the phagosomes are formed once the phagocytic cup detaches from the PM (Nakada-Tsukui et al., 2009; Babuta et al., 2015). For the detachment, EhCaBP3 and EhCaBP5 activate myosin IB, which is involved in the closure of the pseudopodia for the formation and release of the phagosome (Aslam et al., 2012; Kumar et al., 2014). Although in human the dynamin is key for the liberation of the nascent vesicle, in *E. histolytica*, dynamin-like proteins have only been found in the nuclear membrane and mitosomes, but not in endosomes (Jain et al., 2010; Makiuchi et al., 2017). Despite the absence of dynamin in endosomes, other proteins such as ESCRT-III members contribute to the vesicle’s scission. In *in vitro* experiments, using the GUVs model, EhVps20, EhVps32, EhVps24 and EhVps2 participate in the formation and release of ILVs (Avalos-Padilla et al., 2018). Nevertheless, experiments using mutant trophozoites in the ESCRT components would demonstrate the participation of these proteins *in vivo* in the endosomes release.

Alternatively, during 5 to 10 min of phagocytosis, pre-phagosomal vacuoles (PPV) are formed. These structures function as a temporary reservoir for digestive enzymes, transport amoebapores to the phagosome, and contain EhRab5 and EhRab7A (Saito-Nakano et al., 2004; Verma et al., 2015). Dissociation of EhRab5 from PPV promotes the fusion of this compartment with phagosomes of the endocytic pathway (Saito-Nakano et al., 2004).

**Figure 3:**
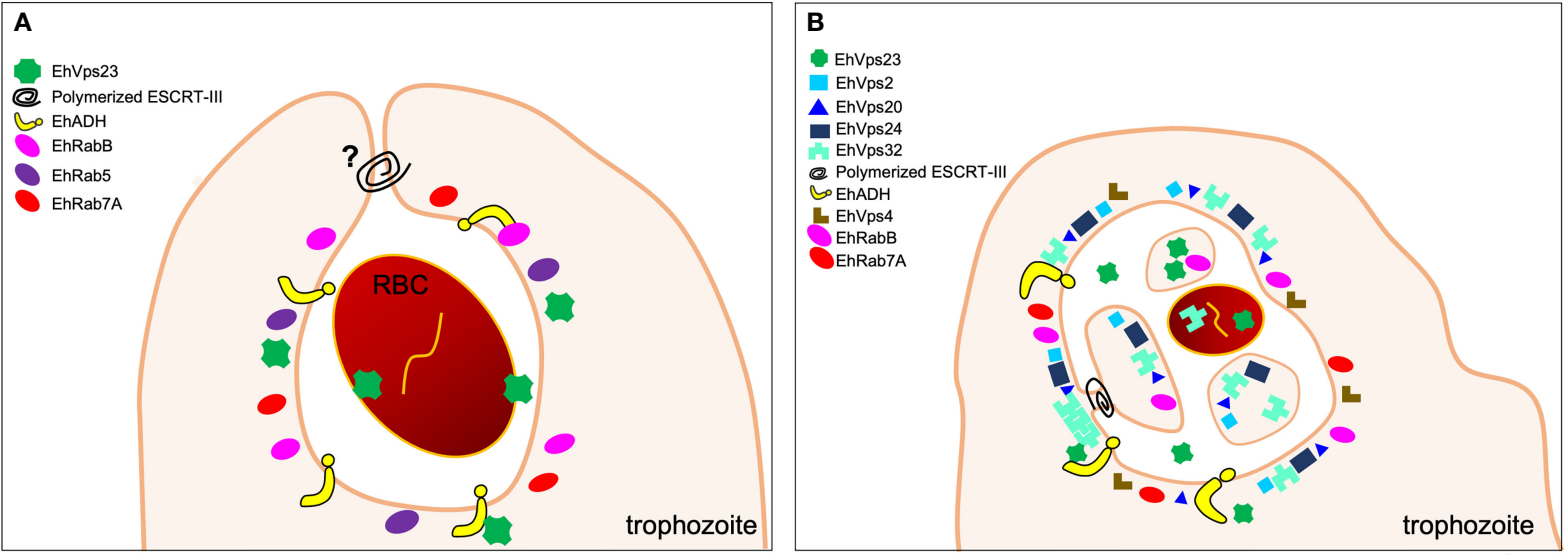
EE and LE/MVBs maturation. **(A)** Once the phagocytic cup detaches from the PM, the phagosomes are formed. For the scission of the nascent vesicles, possibly the polymerized ESCRT-III participates. In EE, several EhRabs and ESCRT-I and ESCRT accessory proteins are localized. **(B)** During the EE transition to LE/MVBs, the ESCRT-III and ESCRT accessory proteins contribute in the ILVs generation. In addition, some EhRabs are localized at LE, suggesting their participation in LE formation.

On the other hand, during the classic pathway for endosome maturation, the first structure formed is the EE and then, the LE or multivesicular bodies (MVBs) (**Figure 3**). In human, Rab5 and Rab7 proteins are specific markers for EE and LE, respectively (Langemeyer et al., 2018). In *E. histolytica*, EhRab5 has also been suggested as a possible EE marker (Verma and Datta, 2019), while EhRab7A and EhRabB have been found in EE or LE (Saito-Nakano et al., 2007; Javier-Reyna et al., 2019; Verma and Datta, 2019) (**Figure 3**). In addition, some proteins of the ESCRT machinery (EhVps2, EhVps4, EhVps20, EhVps23, EhVps24, EhVps32, and EhADH) have been localized in different endosomal structures during erythrophagocytosis (López-Reyes et al., 2010; Bañuelos et al., 2012; Avalos-Padilla et al., 2015; Avalos-Padilla et al., 2018; Galindo et al., 2021) (**Figure 3**). Since EE and LE specific markers are not yet available for *E. histolytica*, the accurate location of ESCRT proteins in the different structures cannot be fully determined yet. Nevertheless, based on the time of erythrophagocytosis, it can be suggested that EhVps23 and EhADH are present in EE (<5 min) (Galindo et al., 2021) (**Figure 3A**). Meanwhile, EhADH has been found in many endosomal vesicles during phagocytosis, from the first contact of the trophozoite with the target cell to the processes of digestion and secretion (Bañuelos et al., 2012; Avalos-Padilla et al., 2015; Galindo et al., 2022). It suggests that this adhesin participates in the entire target cell capture and digestion process. In yeast and human, the ESCRT-III complex participates in the LE maturation (Teis et al., 2008). Accordingly, in *E. histolytica* EhVps2, EhVps4, EhVps20, EhVps24 and EhVps32, and EhADH, are found in LE (>15 min) (**Figure 3B**) (López-Reyes et al., 2010; Bañuelos et al., 2012; Avalos-Padilla et al., 2015; Avalos-Padilla et al., 2018).

### Multivesicular bodies biogenesis

Some LE appear with several ILVs (~50 nm) inside and are referred as MVBs (100-600 nm in diameter), whose content is sent to lysosomal degradation. In other organisms, the ESCRT machinery mediates membrane budding and ILVs formation (Henne et al., 2011; Schuh and Audhya, 2014; Christ et al., 2017), and given the experimental data available, we postulate that in *E. histolytica*, ESCRT complexes and accessory proteins are pivotal for these events that form part of the phagocytosis phenomenon (**Figure 3B**).

The MVBs formation initiates with the ESCRT-0 complex recruitment (Hurley, 2008). In *E. histolytica*, putative sequences of EhVps27 and EhHse1, members of this complex, were *in silico* identified (López-Reyes et al., 2010). However, these proteins lack the VHS (Vps27, Hrs and STAM1), and UIM (ubiquitin-interaction motif) domains, involved in lipid and ubiquitin binding, respectively (López-Reyes et al., 2011). *Dictyostelium discoideum* lacks the typical ESCRT-0 members, but Tom1 performs the ESCRT-0 activity (Blanc et al., 2009). Tom1 proteins harbor a GAT (GGA and Tom1) domain for ubiquitin (Ub) binding, and motifs for clathrin, membrane phospholipids, and Vps23 association (Blanc et al., 2009; Herman et al., 2011; Schuh and Audhya, 2014). In this context, *E. histolytica* has a Tom1 (EhTom1) homolog that, according to our preliminary analysis, possesses an Ub-binding domain, suggesting that it can perform functions similar to those assigned to orthologous members in other organisms.

Next, ESCRT-0 recruits to ESCRT-I. The *E. histolytica* genome contains two genes (*Ehvps23* and *Ehvps37*) of the ESCRT-I complex and their transcription increases after 5 min of erythrophagocytosis (López-Reyes et al., 2010), suggesting that a higher amount of these proteins is necessary for events related to phagocytosis when the MVBs are formed or being formed. The EhVps23 protein is located in MVBs and cytoplasmic vesicles in the basal state, while during phagocytosis it is found in phagosomes and vesicles close to the them (Galindo et al., 2021), strongly suggesting that it is participating in the molecular events performed at this stage of phagocytosis (**Figure 3B**). Moreover, the knock-down of the *Ehvps23* gene causes a lower rate of phagocytosis (50% less), in contrast to trophozoites overexpressing EhVps23, which exhibit a higher rate of phagocytosis (20% more) than the control trophozoites (Galindo et al., 2021; Galindo et al., 2022). In addition, EhVps23 associates to EhADH, EhVps32, LBPA and EhUb, colocalizing in different endosomal structures. As EhADH, EhVps23 could play a role at various points of the endocytic pathway. Another experimental data on the relevance of EhVps23 in phagocytosis is the fact that trophozoites overexpressing EhVps23 migrate five-fold faster than control parasites, in concordance with the low rate of migration exhibited by *Ehvps23*-knocked down trophozoites (Galindo et al., 2022); this, points out to the participation of the ESCRT machinery also in motility, a fundamental event for phagocytosis. In regard to EhVps37, López-Reyes et al. (2010) found an EhVps37D protein lacking the alpha helixes required for ESCRT-I binding (Kostelansky et al., 2007; Galindo et al., 2021). Given the diversity exhibited by *E. histolytica* ESCRT proteins compared to their orthologues (Leung et al., 2008; López-Reyes et al., 2010; López-Reyes et al., 2011; Avalos-Padilla et al., 2018), the possibility of other unidentified ESCRT-I members, cannot be ruled out.

In eukaryotes, after assembly of the ESCRT-I, the ESCRT-II complex is recruited to the endosomal membrane (Hurley, 2008). The *E. histolytica* genome contains the *Ehvps22, Ehvps25* and *Ehvps36* genes that form the ESCRT-II in this parasite (López-Reyes et al., 2011). López-Reyes et al. (2010) also found that *Ehvps36* is downregulated after 5 min stimulation with RBCs. However, more studies at protein level are necessary to elucidate the participation of this complex during phagocytosis.

Then, the ESCRT-II module recruits ESCRT-III proteins that remain in the cytoplasm in an inactive state, which is modified by the binding of Vps25 (ESCRT-II) to Vps20 (ESCRT-III) (Im et al., 2009). ESCRT-III proteins (Vps2, Vps20, Vps24 and Vps32) are regulated through an autoinhibitory switch mechanism that allows a tight control for their assembly. Vps20 suffers a conformational change to generate an open/close configuration that corresponds to an active/inactive state, respectively. Active Vps20 binds to the endosomal membrane, recruits the active Vps32, whereas Vps24 and Vps2 promote polymerization (Bajorek et al., 2009). In *E. histolytica* trophozoites, the four proteins are detected in erythrocytes-containing phagosomes of acidic nature, and in cytoplasmic vesicles at the PM proximity (Avalos-Padilla et al., 2018). ESCRT-III proteins also participate during the formation and release of ILVs within MVBs (**Figure 3B**). Electron microscopy images display EhVps32 forming the helical structures present on LE and phagolysosomes, necessary for endosomal membrane strangulation and ILVs release. The role of the ESCRT-III in ILVs formation was confirmed using the GUVs model. By this system, it was possible to rebuild *in vitro* the whole ESCRT-III machinery and establish the order of assembly of the proteins. First, the active EhVps20 binds to the GUVs membrane and recruits EhVps32, promoting membrane invaginations. EhVps24 allows the detachment of nascent vesicles, forming ILVs; and EhVps2 modulates their size (Avalos-Padilla et al., 2018). The key role of the ESCRT-III complex in phagocytosis is supported by the increased rate of erythrophagocytosis in parasites overexpressing EhVps32 (Avalos-Padilla et al., 2015). In contrast, when *Ehvps20*, *Ehvps24* and *Ehvps32* genes are silenced, a decrease in the erythrophagocytosis rate is observed (Avalos-Padilla et al., 2015; Avalos-Padilla et al., 2018).

Besides to ESCRT-III proteins, EhVps23 (ESCRT-I) and EhADH (ESCRT accessory protein), probably also contribute to the membrane remodelling involved in the formation of ILVs (**Figure 3B**) (Bañuelos et al., 2012; Avalos-Padilla et al., 2015; Avalos-Padilla et al., 2018; Galindo et al., 2021).

In addition to EhADH, the ESCRT machinery needs the help of other accessory proteins, such as the EhVps4-ATPase and EhVta1 (López-Reyes et al., 2010). The EhVps4-ATPase encloses the MIT (Microtubule Interacting and Transport) domain at the N-terminal end, an AAA (ATPase Associated with a variety of Activities) motifs, and the C-terminal end. In human, Vps4-ATPase binds to CHMP2, CHMP4 and CHMP6 from the ESCRT-III, through its MIT domain, and it is probable that this also occurs in *E. histolytica*. EhVps4 has ATPase activity *in vitro* as in other eukaryotes, which depends on the conserved E211 residue (López-Reyes et al., 2010). The EhVps4-ATPase protein localizes around the phagocytosed erythrocytes (**Figure 3B**) when it is overexpressed, and accordingly, mutant trophozoites expressing EhVps4-E211Q exhibit a decrease in their rate of phagocytosis. In yeast and human, Vps4-ATPase enhances its ATPase activity due to Vta1 binding (López-Reyes et al., 2010). The activation of Vps4 by Vta1 implies Vps4 oligomerization and the enhancing of ATP hydrolysis by Vps4 oligomer (Azmi et al., 2006). Vta1 has VSL (Vps4, SBP1 and LIP5) and MIT domains, which allow its interaction with Vps4-ATPase and ESCRT-III proteins, respectively (Yeo et al., 2003). *E. histolytica* has the *Ehvta1* gene, which is transcribed in basal conditions and phagocytosis (López-Reyes et al., 2010; López-Reyes et al., 2011). So far, bioinformatics analysis has not revealed the presence of VSL and MIT domains in EhVta1; however, we cannot discard interactions with EhVps4-ATPase and ESCRT-III members by other regions. Further research will unveil if the activity of EhVps4 depends on or is potentiated by EhVta1.

On the other hand, in mammalian cells, an alternative ubiquitin-independent MVBs formation pathway, in which Alix and ESCRT-III proteins are involved (Dores et al., 2012), has been reported. In human, PAR1-activated receptor directly binds to Alix; then, Alix recruits CHMP4B and the rest of the ESCRT-III subunits, in an ubiquitin-independent manner (Dores et al., 2012). This also could be happening in *E. histolytica*, suggesting the participation of EhADH in MVBs formation, since it acts as an erythrocyte receptor by its adherence domain, whereas by its Bro1 domain, it recruits EhVps32.

Overall, these data point out to the relevance of the concerted interactions among *E. histolytica* ESCRT proteins, as in other eukaryotes, for the membrane remodeling during the endocytic pathway (**Figure 3B**). In *E. histolytica*, some of these interactions need to be experimentally validated to confirm their role during phagocytosis.

### Lysosomal degradation

**Figure 4:**
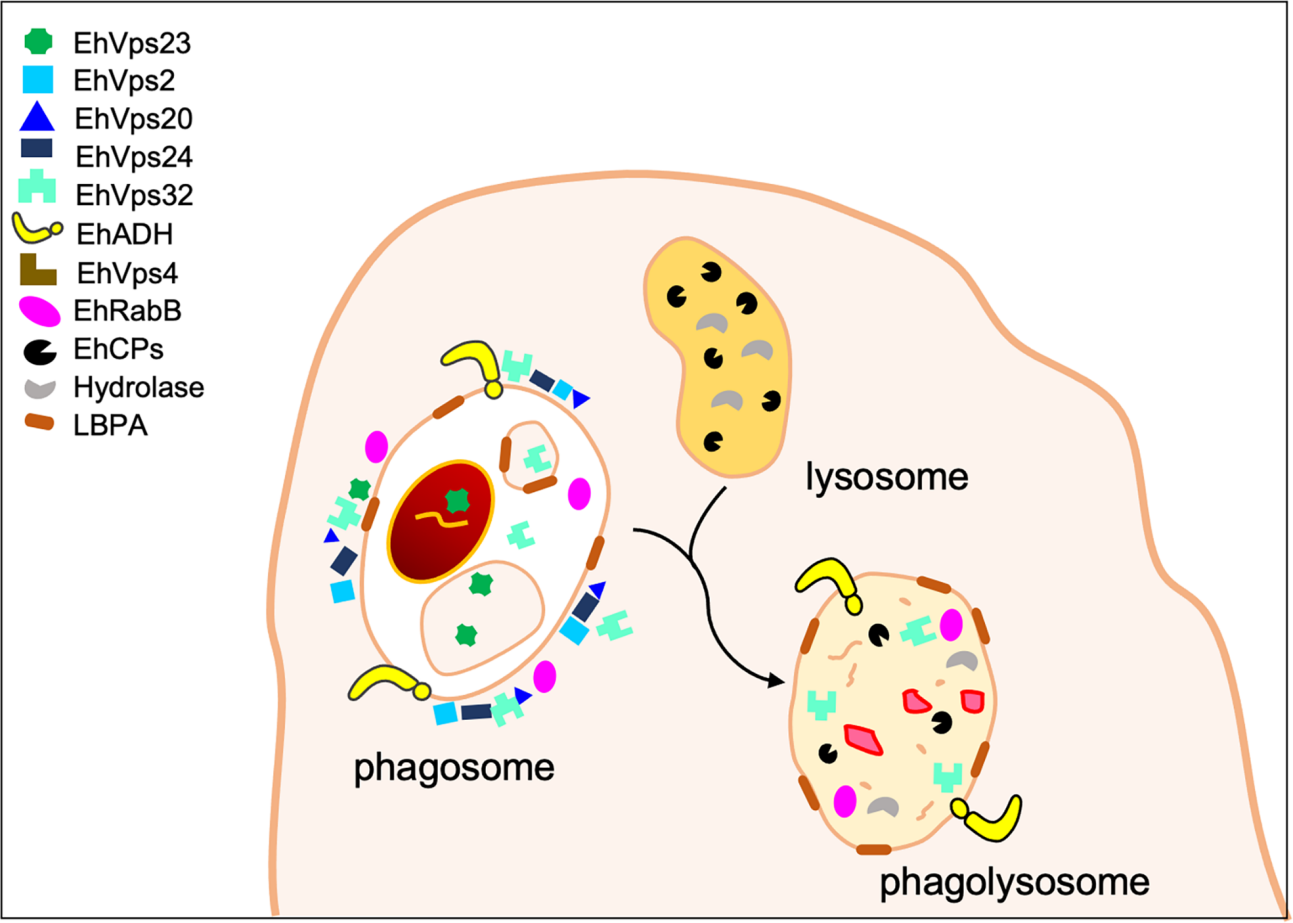
Lysosomal degradation. Particularly during phagocytosis, LE/MVBs are also called late phagosomes. They fused to lysosomes for generating phagolysosomes. Lysosomes are enriched in hydrolases, which contribute to degrade molecules in the phagolysosomes. In the latter, ESCRT-III and ESCRT accessory proteins, together with EhRabB and LBPA, are also present.

The endosome maturation culminates with the phagosome fusion to the lysosome, forming the phagolysosome, which contains hydrolytic enzymes (EhCP1, EhCP2, EhCP4, EhCP5, amoebapores, phospholipases, dipeptidyl aminopeptidase, β-hexosaminidase, and lysozymes) (Long-Krug et al., 1985; Nickel et al., 1998; Flockenhaus et al., 2000; Que et al., 2002; Andrä et al., 2003; Furukawa et al., 2012); membrane receptors (Gal/GalNAc lectin) (Petri et al., 2002); and proteins related to cytoskeletal rearrangements and vesicular trafficking, such as EhRabs (**Figure 4**) (Marion et al., 2005; Okada et al., 2006; Saito-Nakano et al., 2007; Verma and Datta, 2017). In this step, the role of Rabs is crucial to the protein transport and their degradation in lysosomes. Particularly, EhRab7B regulates EhCPs and holo-transferrin transport to lysosomes (Saito-Nakano et al., 2007; Smith and Guillen, 2010; Verma et al., 2015). Besides, the expression of an EhRab7B mutant reduces phagocytosis, lysosome acidification, and intracellular EhCPs activity (Saito-Nakano et al., 2007). The overexpression of EhRab35 increases the number of lysosomal compartments and the degradation of RBCs (Verma and Datta, 2017; Constantino-Jonapa et al., 2020). All these experimental results give strong evidence on the role of Rab proteins in different steps of phagocytosis.

In human, the ESCRT machinery plays an additional role on endolysosomal organelles, responding to and promoting the repair of damaged or perforated membranes (Chen et al., 2019). Alix co-localizes with CHMP4A and other ESCRT-III components on endolysosomes after acute damage produced by L-leucyl-L-leucine O-methyl ester. The silencing of *tsg101* and *alix* genes, from the ESCRT-I complex and ESCRT accessory proteins, respectively, prevents the recruitment of ESCRT-III members to the lysosomes, reducing the repair of these structures (Skowyra et al., 2018). Moreover, using live cell imaging, it was demonstrated that ESCRTs respond to small perforations in endolysosomal membranes and enable compartments to recover from limited damage (Radulovic et al., 2018; Skowyra et al., 2018). The need to protect the endolysosomal integrity has broad implications for many situations, perhaps most critically in highly phagocytic cells as *E. histolytica*, that internalizes and processes substantial loads of disruptive material. In this parasite, EhVps32 and EhADH as part of the ESCRT-III and ESCRT accessory proteins, respectively, as well as the molecules that bind to ESCRT components, such as EhRabB and LBPA, are present in the phagolysosomes during RBCs degradation (**Figure 4**) (Bañuelos et al., 2012; Avalos-Padilla et al., 2015; Castellanos-Castro et al., 2016a; Javier-Reyna et al., 2019). Besides, the ESCRT-III proteins exhibit a propensity to assemble into spirals on highly curved membranes, and surround, constrict and ultimately close vesicles (Wollert et al., 2009; Henne et al., 2012; Avalos-Padilla et al., 2018). Thus, we hypothesize that the ESCRT-III proteins may repair damaged membranes on phagolysosomes, resealing wounds. Here, the EhADH protein, together with EhVps23, could be contributing to the recruitment of ESCRT-III components, since both bind to EhVps32.

## Cargo recycling

### Plasma membrane recycling

**Figure 5:**
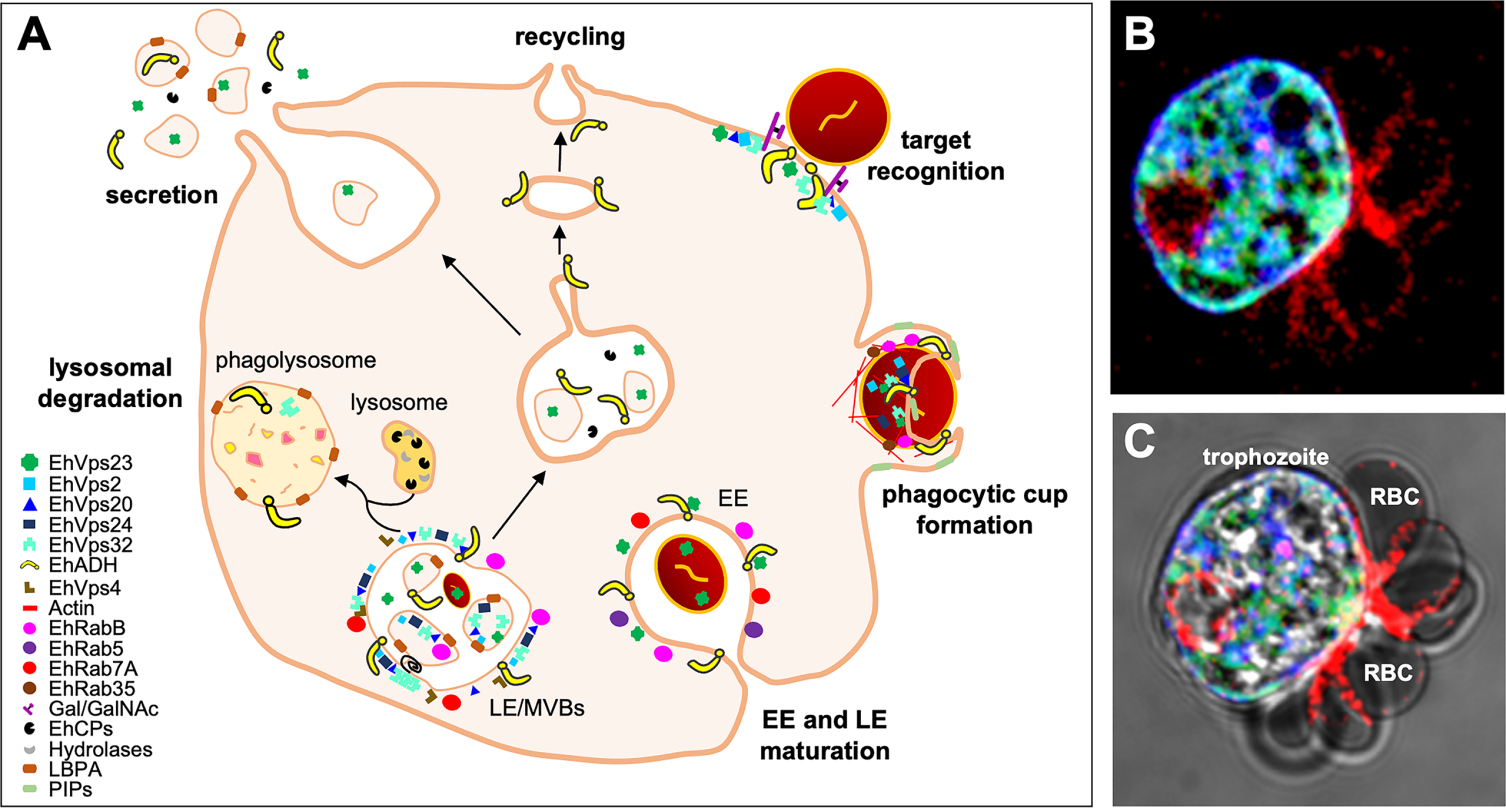
The ESCRT machinery in the endosomal compartments during phagocytosis. **(A)** Phagocytosis begins with the RBCs recognition. RBCs are internalized through the phagocytic cup to generate EE that eventually will mature into LE/MVBs. These structures fuse to lysosomes for producing phagolysosomes, where the molecules are finally degraded. In parallel, some proteins as receptors are recycled to the PM to be reused for new endocytosis rounds. On the other hand, in order to modulate the communication with other trophozoites or for damaging host cells, *E*. *histolytica* secretes molecules to the extracellular medium, such as ESCRT proteins, EhCP112, amoebapores and LBPA, among others. In general, several ESCRT members have been detected in different types of endosomes or vesicles, suggesting their active participation during the endocytic pathway, recycling and secretion. **(B, C)** Immunofluorescence assays and confocal microscopy of trophozoites ingesting RBCs. Images show EhADH (green), EhVps32 (blue) and actin (red) at the PM, particularly at the contact site with RBCs. In addition, these proteins are also located around ingested RBCs and in some intracellular structures. **(C)** Overlapping image of fluorescence channels plus phase contrast evidencing the trophozoite and RBCs morphology.

After endocytosis, transmembrane cargo reaches endosomes, where it encounters complexes dedicated to opposing functions: degradation and recycling (Schuh and Audhya, 2014; Scott et al., 2014). There is scarce data about the molecules involved in the recycling pathway in *E. histolytica* and even less, regarding to the ESCRT’s role in this event (**Figure 5A**). By the way, Rab proteins are key membrane trafficking organizers that could be contributing to integrate and coordinate the ESCRT complexes towards the recycling pathway (Arlt et al., 2015). Rab8 and Rab11, together with their effector proteins, coordinate the control of proteins trafficking from the trans-Golgi to the PM in mammalian cells (Chen et al., 1998). In *E. histolytica*, the transport of receptors to the PM is necessary for binding to host cells. EhRab8A primarily resides in the endoplasmic reticulum (ER) and participates in phagocytosis. Its down-regulation by small antisense RNA-mediated transcriptional gene silencing remarkably reduces adherence and phagocytosis of erythrocytes, bacteria and carboxylated latex beads. Moreover, the surface expression of several proteins presumably involved in *E. histolytica* target recognition, is reduced in the EhRab8A in italics gene-silenced strain, indicating that EhRab8A regulates transport of surface receptors for the prey from the ER to the PM (Hanadate et al., 2016).

EhRab11A translocates to the cell surface upon starvation, and it has been implicated in the transport of cyst wall components such as enolase, to the cell surface *via* actin filaments (Herrera-Martínez et al., 2013). EhRab11B is associated with non-acidified vesicles considered as recycling compartments, and regulates the secretion of EhCP1, EhCP2 and EhCP5 in a brefeldin insensitive manner (Mitra et al., 2007).

EhRabB and the actin cytoskeleton participate in the transport of the EhCPADH complex towards the trophozoite PM (Javier-Reyna et al., 2019). As we have mentioned throughout this work, EhADH participates in several points of the endocytic pathway, where it has been localized with proteins of the ESCRT machinery. Likewise, the presence of some of ESCRT components close to the PM, such as EhVps2, EhVps20, EhVps23 and EhVps32, suggests that these proteins may participate in the mobilization of EhADH to the PM to interact with RBCs (**Figure 5**).

The role of ESCRT in cargo recycling, and its relationship with EhRabs have not been reported yet in *E. histolytica*. In *Trypanosoma brucei*, TbRab28 colocalizes with the ESCRT-I component Vps23 and is required for the turnover of internalized surface glycoproteins (Lumb et al., 2011). Furthermore, the *T. brucei* ubiquitylated invariant surface glycoprotein (ISG65) is rescued from lysosome delivery by the ESCRT accessory protein TbVps4-ATPase, to be recycled to the cell surface. In addition, the phosphoinositide-dependent binding of the ESCRT-III component TbVps24, affects the ISG65 traffic and accelerates its surface pool depletion. TbVps24 localizes to TbRab7 late endosome, and binds PI(3,5)P2 (Umaer and Bangs, 2020). Authors propose a model in which *T. brucei* ESCRT-III and ESCRT accessory components operate at two sites, one PI(3,5)P2 -dependent (degradation) and one PI(3,5)P2 -independent (recycling), to regulate ISG65 homeostasis. In this context, considering that late ESCRT proteins such as EhVps24 and EhVps4-ATPase are expressed by *E. histolytica*, we hypothesize that these proteins could exhibit a similar role during protein recycling in trophozoites.

### Retromer

Another alternative recycling pathway is driven by the retromer, a complex of proteins that recycle transmembrane receptors from endosomes to the trans-Golgi network (Seaman, 2012). The ESCRT and retromer pathways drive opposing endosomal functions that must somehow coexist; therefore, understanding the segregation of ESCRT and retromer domains on the endosome may be particularly relevant to understanding endosome function. In *E. histolytica*, the retromer is formed by EhVps26, EhVps29, EhVps35 (Loftus et al., 2005; Nakada-Tsukui et al., 2005), EhSNX1 and EhSNX (Batra et al., 2021), and has been associated with the EhCP’s pool maintenance (Nakada-Tsukui et al., 2005). The association among EhVps26, EhVps29, and EhVps35 was evidenced by immunoprecipitation and mass spectrometric analysis (Nakada-Tsukui et al., 2005). By immunoprecipitation experiments using α-EhVps23 antibodies and mass spectrometry analysis, our group detected EhVps26 and EhVps35 as interacting partners of EhVps23 (Galindo et al., 2022).

In *E. histolytica*, the retromer associates with EhRab proteins. EhRab7A regulates the recycling of a non-identified EhCP receptor from the phagosomes to the trans-Golgi network (Saito-Nakano et al., 2004; Nakada-Tsukui et al., 2005; Nozaki and Nakada-Tsukui, 2006). EhRab7A binds to a sequence rich in charged amino acids located at the C-terminal end of EhVps26. EhRab7A overexpression produces the reduction of EhCPs activity, but EhVps26 overexpression restores it (Nakada-Tsukui et al., 2005; Nozaki and Nakada-Tsukui, 2006). Instead, the EhVps29 overexpression leads to a reduction of intracellular EhCPs activity (Srivastava et al., 2017). Altogether, these data suggest a role for the retromer as a machinery essential for the restitution of EhCP receptors, as it has been demonstrated in other eukaryotes (Wang et al., 2018), most likely *via* protein trafficking.

Despite the relative absence of evidences around the relationship among the retromer and the ESCRT machinery, possible regulatory interactions should exist to balance degradative and recycling functions, as it has been reported for *Caenorhabditis elegans*, in which segregating microdomains are enriched in the retromer from those enriched in ESCRT-0 for maintaining the required balance between recycling and degradation activities within the endosome pathway (Norris et al., 2017). Since endosomes are a mosaic of functional arrays consisting of transmembrane cargo, lipids, and peripheral membrane proteins, it is expected that these components segregate and exhibit a dynamic performance in trophozoites, changing over space and time. Thus, as an endosome matures, it will change in structure and function, determining the final fate of cargo molecules.

Overall, findings suggest that recycling pathways are cross-regulated by ESCRT to maintain an appropriate balance of each activity within a given endosome and its sorting. Further research will confirm the role of the ESCRT machinery in proteins recycling in *E. histolytica*.

## ESCRT proteins and secretion

During its pathogenic mechanism, *E. histolytica* secretes several molecules to reach the target cell and initiate invasion or we can speculate that also to communicate with other trophozoites. Likewise, cytolysis of target cells is carried out by cytolytic proteins secreted to the extracellular medium, including EhCPs (Que et al., 2002) and amoebapores (Zhang et al., 2004). Hydrolytic enzymes secretion is led by a specific interaction ligand-receptor, conducting to a dynamic vesicular transport and cytoskeletal rearrangement (**Figure 5A**) (Nozaki and Nakada-Tsukui, 2006).

Other molecules that have been experimentally probed as secreted products by this parasite are: prostaglandin E2, EhNPC1 and EhNPC2, and some ESCRT components, including EhVps23 and EhADH (**Figure 5A**) (García-Rivera et al., 1999; Ocádiz et al., 2005; Sato et al., 2006; Lejeune et al., 2011; Bolaños et al., 2016; Galindo et al., 2022). Particularly, ESCRT components are secreted in vesicles (Galindo et al., 2022), which probably carry molecules that participate in the prey capture or in cell-cell communication (**Figure 5A**). Regarding to this, in mammalian cells, ILVs from MVBs also modulate intercellular communication when they are targeted to the PM for being secreted as exosomes. Here, an Alix- and ESCRT-III–dependent pathway promotes the sorting and delivery of tetraspanins to exosomes (Larios et al., 2020). However, in extracellular vesicles (ECVs) secreted by *E. histolytica*, tetraspanins have not been found yet (Sharma et al., 2020), although EhADH is there (**Figure 5A**) (Galindo et al., 2022). In addition, as mentioned above, EhADH provides an additional pathway for MVBs formation (Avalos-Padilla et al., 2015), and, by this route, this ESCRT accessory protein could control the targeting of exosomal proteins.

The ESCRT-III proteins recruitment to the endosomes could occur independently of other ESCRTs (Avalos-Padilla et al., 2018), but might require LBPA, as it has been reported in human and yeast (Larios et al., 2020). The ESCRT-related proteins that have been found interacting with LBPA in *E. histolytica*, include EhVps23 and EhADH (Castellanos-Castro et al., 2016b; Castellanos-Castro et al., 2016a; Galindo et al., 2021).

**Figure 6:**
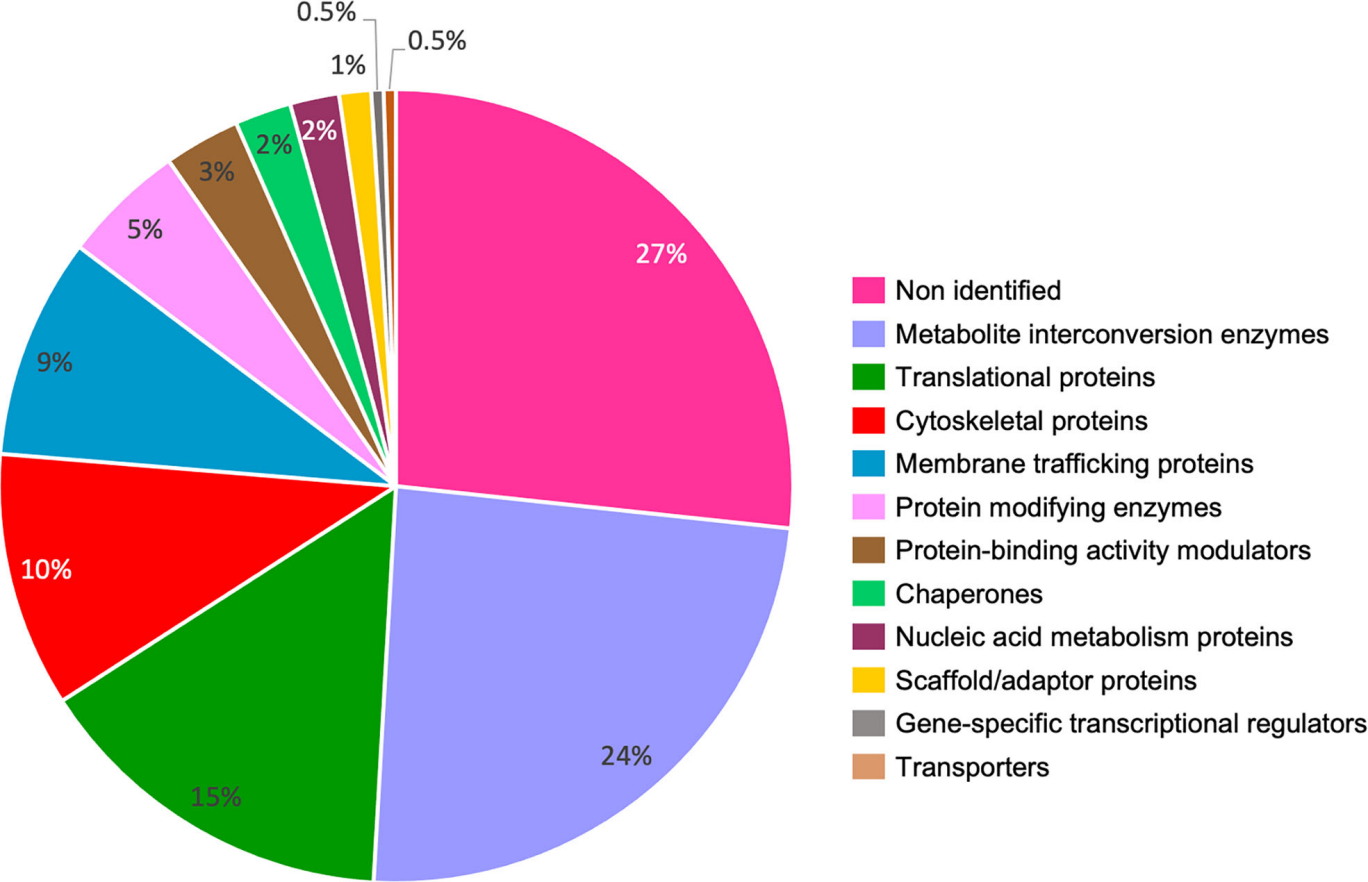
*E. histolytica* secreted proteins that associate to LBPA. Secreted proteins from cultured trophozoites were immunoprecipitated with an α-LBPA antibody as previously reported (Castellanos-Castro et al., 2016a). To remove the background, secreted products were pre-cleared with recombinant protein G (rProtein-G) agarose ON, previous to the incubation with the specific antibody (α-LBPA). The LBPA-bound proteins were identified by mass spectrometry (LC-ESI-HDMSE) in the Proteomics Unit of LaNSE (National Laboratory of Experimental Services) at CINVESTAV. Then, theoretical astringent conditions were performed to eliminate proteins with low confidence values. Proteins (221) were organized by biological processes using the Panther server.

By a proteomic approach, 496 secreted proteins from cultured trophozoites were immunoprecipitated using an α-LBPA antibody, followed by mass spectrometry analysis (LC-ESI-HDMSE). After theoretical astringent conditions, only 221 proteins exhibited high confidence values. The graph (**Figure 6**) shows that the highest percentages of proteins obtained correspond to the non-identified category (27%), followed by metabolite interconversion enzymes (24%), translational proteins (15%), cytoskeletal proteins (10%), and proteins involved in membrane trafficking (9%). From these groups, we enlist 44 proteins related to endocytosis, vesicular trafficking and cytoskeleton (**Table 2**), and the rest of the proteins in the **Supplementary Table I**.

**Table 2:** *E. histolytica* secreted proteins that associate to LBPA and related to vesicular trafficking and cytoskeleton.

Access number	Protein	Putative function
EHI_105270	Receptor mediated endocytosis protein	Endocytosis
EHI_201510	Clathrin heavy chain, putative^+^	Endocytosis
EHI_201710	Clathrin heavy chain, putative^+^	Endocytosis
EHI_201940	Clathrin heavy chain, putative^+^	Endocytosis
EHI_073470	EhArf1	Vesicular trafficking
EHI_059670	Rab family GTPase	Vesicular trafficking
EHI_146510	Rab family GTPase	Vesicular trafficking
EHI_164900	Rab family GTPase	Vesicular trafficking
EHI_143650	EhRabC3	Vesicular trafficking
EHI_096220	EhRabC4	Vesicular trafficking
EHI_170390	EhRabC8	Vesicular trafficking
EHI_108610	EhRab1A	Vesicular trafficking
EHI_081330	EhRab7A	Vesicular trafficking
EHI_199820	EhRab8A*	Vesicular trafficking and phagocytosis
EHI_127380	EhRab8B	Vesicular trafficking
EHI_167060	EhRab GDP dissociation inhibitor alpha	Vesicular trafficking
EHI_058090	EhRas family GTPase	Vesicular trafficking
EHI_197840	Rho family GTPase	Vesicular trafficking
EHI_187110	Rho GTPase activating protein	Vesicular trafficking and cytoskeleton
EHI_147570	EhRho GDP exchange inhibitor	Vesicular trafficking
EHI_148190	GTP-binding nuclear protein	Vesicular trafficking
EHI_041950	EhVps35	Vesicular trafficking
EHI_160900	EhVps45A	Vesicular trafficking
EHI_008730	EhSec23, component of COPII	Vesicular trafficking
EHI_048310	EhSec24C, component of COPII	Vesicular trafficking
EHI_118050	Coatomer alpha subunit	Vesicular trafficking
EHI_124460	AP-1 complex subunit mu-2	Vesicular trafficking
EHI_182900	Actin	Cytoskeleton
EHI_198930	Actin, putative	Cytoskeleton
EHI_111050	Actin-like protein, putative	Cytoskeleton
EHI_052780	F-actin bound-C domain-containing protein	Cytoskeleton
EHI_005020	F-actin-capping protein subunit beta	Cytoskeleton
EHI_104390	Actin binding protein	Cytoskeleton
EHI_186770	Actin-binding protein, cofilin/tropomyosin family	Cytoskeleton
EHI_186840	Actin-binding protein, cofilin/tropomyosin family	Cytoskeleton
EHI_045000	Arp2/3 complex subunit	Cytoskeleton
EHI_174910	Arp2/3 complex subunit 3	Cytoskeleton
EHI_152660	Arp2/3 complex 20 kDa subunit	Cytoskeleton
EHI_199690	Arp2/3 complex 34 kDa subunit	Cytoskeleton
EHI_105210	F-BAR domain-containing protein	Cytoskeleton
EHI_142190	WD repeat protein 2	Cytoskeleton
EHI_136150	Adenylyl cyclase-associated protein	Cytoskeleton
EHI_080740	Filopodin	Cytoskeleton
EHI_176140	Profilin	Cytoskeleton

Secreted proteins from trophozoites were immunoprecipitated with an α-LBPA antibody, as previously reported (Castellanos-Castro et al., 2016a). The LBPA-bound proteins were identified by mass spectrometry (LC-ESI-HDMSE) in the Proteomics Unit of LaNSE at CINVESTAV, and restricted to theoretical astringent conditions, resulting 221 proteins with high confidence values, from which, 44 proteins are related to endocytosis, vesicular trafficking and cytoskeleton.

Most of proteins were reported by Loftus et al. (2005).

*It is also mentioned in Hanadate et al. (2016).

^+^Proteins only reported in the AmoebaDB and Uniprot databases.

Next, we performed a gene ontology (GO) enrichment analysis to the 221 secreted proteins immunoprecipitated with α-LBPA, to gain insights into the cellular functions and biological processes related to them (**Figure S1**). Regarding the cellular component terms, the predominant categories correspond to membrane-related and cytoskeleton proteins; meanwhile, the molecular function terms referred to binding, cytoskeleton, and catalytic activity. The analysis for the biological processes in which proteins could be involved, pointed out that presumably, they are participating in metabolic events, translation and cytoskeleton organization (**Figure S1**). In conclusion, this analysis evidences that most of the secreted proteins interacting with LBPA are mainly cytoskeletal proteins or related to them, revealing that cytoskeleton results fundamental for the transport and secretion of proteins.

Of note, the assays were carried out in basal conditions and possibly for this reason, in the proteome, EhADH and EhVps23 were not detected. Also, it is feasible that the amount of proteins or their high susceptibility to degradation, derived in undetectable products. Further experiments under erythrophagocytosis conditions should be performed to confirm the interaction of these ESCRT proteins with LBPA.

## Concluding remarks

In this review, we recapitulated the known interactions between distinct ESCRT complexes’ subunits, and virulence factors and molecules from other nature in the *E. histolytica* protozoan. Some approaches used at this moment to study the ESCRT machinery include the bioinformatical analysis of its members, proteins modelling, molecular docking, the generation and phenotypic characterization of trophozoite mutants (summarized in **Table 3**), the *in vitro* reconstruction of GUVs, and importantly, the identification of new partners of ESCRT components by proteomic approaches. However, there is a challenge to extend this knowledge to the interactions required for the successful attack of the amoeba, as a biological entity that reaches molecules, cells and organs from the host.

**Table 3:** Trophozoites phenotypes produced by alterations in some ESCRT components.

Protein	Protein alteration	Phenotype	References
EhVps23	Overexpression	Increased rate of growth, erythrophagocytosis, migration and tissue invasion	(Galindo et al., 2022)
	Knock-down	Decreased rate of growth, erythrophagocytosis and migration. It is eventually lethal.	(Galindo et al., 2021; Galindo et al., 2022)
EhVps20	Knock-down	Decreased rate of erythrophagocytosis	(Avalos-Padilla et al., 2018)
EhVps24	Knock-down	Decreased rate of erythrophagocytosis	(Avalos-Padilla et al., 2018)
EhVps32	Overexpression	Increased rate of erythrophagocytosis	(Avalos-Padilla et al., 2015)
	Knock-down	Decreased rate of erythrophagocytosis	(Avalos-Padilla et al., 2015)
EhADH	Overexpression	Increased rate of adherence and erythrophagocytosis	(Bañuelos et al., 2012)
	Bro1 domain overexpression	Decreased rate of erythrophagocytosis	(Bañuelos et al., 2012)
EhVps4	Inactive form overexpression (EhVps4-E211Q)	Decreased cytopathic activity, erythrophagocytosis and tissue invasion	(López-Reyes et al., 2010)

During host colonization, the uptake of cargo or the prey is central for trophozoites survival, and depends on endocytosis. This process demands a dynamic membrane remodelling and an active transport of molecules, which is finely regulated for the concerted performance of proteins. In this event, endosomes formation allows the sorting and internalization of a wide variety of molecules, and even whole cells, with the inherent biogenesis and trafficking of vesicles. Here, several molecules such as EhADH, EhRabs, actin and LBPA, participate. The interaction among these molecules inside and outside of trophozoites, have been revealed by both, bioinformatics and experimental strategies, where proteomics has elucidated meaningful data. In this review, we report proteins that are secreted to the extracellular environment and interact with LBPA, most of them related to endocytosis, membrane trafficking and cytoskeleton. Moreover, these molecules could act in an orchestrated fashion with the assistance of cellular machineries (i.e., ESCRT), resulting in a functional integrated system.

The ESCRT machinery comprises an evolutionary conserved group of specialized proteins that have shed light on the mechanisms underlying protein sorting, MVBs biogenesis, membrane trafficking and cell signalling. Therefore, in this work, we put forward the putative role of the ESCRT system as a platform contributing for membrane remodelling, molecular transport to different compartments, endosome maturation, recycling and secretion. Remarkably, the ESCRT accessory protein EhADH emerges as a scaffold protein that assists the ESCRT machinery functions for connecting molecules and events along the endocytic pathway.

Hence, *E. histolytica* is a suitable model of study for pleomorphism and phagocytic capacities, which results very useful for a comprehensive understanding of the biomedical implications of amoebiasis and its potential strategies of control.

## Author contributions

CB, AB, RJ-R, AG, and EO contributed to the conception and design of the review. RJ-R performed the proteomic analysis. AG designed the table and figures. All authors contributed to manuscript revision, and read and approved the submitted version.

## Funding

This work was supported by the National Council for Science and Technology (CONACYT) of Mexico (grants: A1-S8380 for EO and 284477 for AB), and RJ-R received a CONACYT Postdoctoral Fellowship.

## Conflict of interest

The authors declare that the research was conducted in the absence of any commercial or financial relationships that could be construed as a potential conflict of interest.

The handling editor declared a shared affiliation with the authors at the time of review.

## Publisher’s note

All claims expressed in this article are solely those of the authors and do not necessarily represent those of their affiliated organizations, or those of the publisher, the editors and the reviewers. Any product that may be evaluated in this article, or claim that may be made by its manufacturer, is not guaranteed or endorsed by the publisher.
