# Supplementary material for: Molecular interplays of the Entamoeba histolytica endosomal sorting complexes required for transport during phagocytosis
Source: Front Cell Infect Microbiol. 2022 Oct 27;12:855797. doi: 10.3389/fcimb.2022.855797 (PMC9647190; doi:10.3389/fcimb.2022.855797)
Supplement: Supplementary file 1 [file DataSheet_1.docx]

Supplementary Material

**Supplementary Table 1.** *E. histolytica* secreted proteins that associate to LBPA.

| **Access number** | | **Putative protein** |
| --- | --- | --- |
| **AmoebaDB** | **Uniprot** |  |
| EHI_000440 | C4LZ95 | UTP-glucose-1-phosphate uridylyltransferase |
| EHI_000730 | C4LZC2 | Pyrophosphate-fructose 6-phosphate 1-phosphotransferase |
| EHI_004550 | C4LYR5 | Uncharacterized protein |
| EHI_005060 | Q869B1 | Fe-hydrogenase |
| EHI_005100 | C4M0A4 | Eukaryotic translation initiation factor 2 alpha subunit |
| EHI_005150 | C4M0A8 | Uncharacterized protein |
| EHI_006810 | C4M0F4 | 14-3-3 domain-containing protein |
| EHI_008380 | C4M0L4 | Aminopeptidase |
| EHI_009530 | Q24801 | Pyruvate, phosphate dikinase |
| EHI_010570 | C4LYU4 | Uncharacterized protein |
| EHI_011210 | C4M7D4 | Elongation factor 1-alpha |
| EHI_012480 | B1N2I7 | 60S ribosomal protein L10a |
| EHI_012580 | C4LTP4 | Uncharacterized protein |
| EHI_014030 | Q24858 | Proton-translocating NAD(P) (+) transhydrogenase |
| EHI_014110 | B1N4V3 | 60S ribosomal protein L35a |
| EHI_015120 | C4MBQ9 | Leucine rich repeat protein |
| EHI_015300 | C4M0W8 | DUF1394 domain-containing protein |
| EHI_019600 | C4MA46 | Pelota protein |
| EHI_020270 | C4M7T9 | UBA-e1-C domain-containing protein |
| EHI_020300 | C4M7U2 | Ribosomal protein L15 |
| EHI_021360 | C4M051 | Uncharacterized protein |
| EHI_023110 | C4LUM3 | PKS-ER domain-containing protein |
| EHI_023470 | C4LUQ6 | Oxysterol binding protein |
| EHI_024230 | C4M0H9 | Cysteine synthase A, putative |
| EHI_025360 | C4M323 | 14-3-3 domain-containing protein |
| EHI_026480 | C4M5W1 | (2r)-phospho-3-sulfolactate synthase |
| EHI_029050 | C4M4W9 | Long-chain-fatty-acid-CoA ligase |
| EHI_029350 | C4MAX2 | Uncharacterized protein |
| EHI_029620 | C4M7W2 | Aldose reductase |
| EHI_030610 | B1N591 | MIB/HERC2 domain-containing protein |
| EHI_030750 | C4M1E4 | PPi-type phosphoenolpyruvate carboxykinase 2 |
| EHI_033250 | C4M6Y2 | Polyadenylate-binding protein |
| EHI_035370 | C4LXW9 | Uncharacterized protein |
| EHI_040420 | C4M2H1 | Uncharacterized protein |
| EHI_042170 | C4M4H1 | Aminoacyl-histidine dipeptidase |
| EHI_043010 | Q24802 | H(+)-transporting two-sector ATPase |
| EHI_044740 | C4LT93 | UMP-CMP kinase |
| EHI_044970 | Q9NH04 | Malic enzyme |
| EHI_047800 | C4M7X1 | Uncharacterized protein |
| EHI_047810 | C4M7X2 | Lysine-tRNA ligase |
| EHI_048740 | C4M2Y7 | PNP-UDP-1 domain-containing protein |
| EHI_049620 | Q8MU40 | Nitrogen fixation protein NifU |
| EHI_050130 | C4LTQ0 | Ribosomal-L14e domain-containing protein |
| EHI_050280 | C4LTQ5 | 40S ribosomal protein S3a |
| EHI_050550 | C4LTS8 | WD-repeats-region domain-containing protein |
| EHI_050780 | C4LTU8 | U1snRNP-specific protein |
| EHI_050940 | C4LTW4 | Phosphoglycerate mutase (2,3-diphosphoglycerate-independent) |
| EHI_051060 | C4LTX6 | Pyruvate:ferredoxin oxidoreductase |
| EHI_052860 | C4M3S5 | Heat shock protein 70 |
| EHI_053160 | C4M3D3 | Uncharacterized protein |
| EHI_056480 | C4M6Z7 | Triosephosphate isomerase |
| EHI_060740 | C4MBE2 | EF-hand calcium-binding domain containing protein |
| EHI_068200 | C4LU13 | 60S ribosomal protein L31 |
| EHI_068250 | C4LU18 | Adenosyl homocysteinase |
| EHI_068510 | C4LU42 | Uncharacterized protein |
| EHI_068660 | C4LU56 | Ribosomal-L18-c domain-containing protein |
| EHI_069320 | C4LWM0 | C2 domain containing protein |
| EHI_070720 | C4M5S9 | Inositol-3-phosphate synthase |
| EHI_073460 | C4LXL0 | Diadenosine tetraphosphate synthetase |
| EHI_073650 | Q6SCK6 | Mitogen-activated protein kinase |
| EHI_077270 | C4M2T6 | rRNA biogenesis protein RRP5 |
| EHI_079860 | C4M2K7 | Methionyl-tRNA synthetase |
| EHI_080260 | C4M8Y1 | Uncharacterized protein |
| EHI_081410 | C4M5A0 | 40S ribosomal protein SA |
| EHI_083250 | C4M758 | NAC-A/B domain-containing protein |
| EHI_083270 | C4M760 | Ubiquitin-like domain-containing protein/EhUBI1 |
| EHI_085980 | C4M353 | Glycosyltransferase |
| EHI_086120 | C4M367 | 40S ribosomal protein S18 |
| EHI_088020 | C4M6R1 | Alcohol dehydrogenase |
| EHI_092490 | C4LV11 | Uncharacterized protein |
| EHI_092640 | C4LV24 | Seryl-tRNA synthetase |
| EHI_093900 | C4LYI1 | DEAD/DEAH box helicase |
| EHI_094100 | C4LYJ7 | Galactokinase |
| EHI_096420 | C4LWF5 | LIM zinc finger domain containing protein |
| EHI_096830 | C4LWJ2 | Alpha-1,4 glucan phosphorylase |
| EHI_098280 | C4LXB1 | 14-3-3 domain-containing protein |
| EHI_098290 | Q27647 | Phosphotransferase |
| EHI_098560 | Q27648 | Phosphotransferase |
| EHI_098570 | C4LXD7 | Fructose-1,6-bisphosphate aldolase |
| EHI_098800 | C4M882 | Epimerase domain-containing protein |
| EHI_099700 | C4M6H3 | NAD(FAD)-dependent dehydrogenase |
| EHI_101240 | C4M8B5 | Uncharacterized protein |
| EHI_103310 | C4LVH6 | 60S ribosomal protein L7a |
| EHI_103430 | C4LVI7 | Cortexillin II |
| EHI_103730 | C4LVL5 | Threonyl-tRNA synthetase |
| EHI_103860 | C4LVM8 | Uncharacterized protein |
| EHI_104330 | C4LZ45 | Heat shock protein70 |
| EHI_104560 | B1N341 | Cortexillin |
| EHI_104630 | C4LZ72 | Filamin 2 |
| EHI_105060 | C4M111 | Geranylgeranyl pyrophosphate synthase |
| EHI_106090 | C4M384 | 1,4-alpha-glucan branching enzyme |
| EHI_107210 | C4M162 | NADP-dependent alcohol dehydrogenase |
| EHI_110120 | C4LU66 | Phosphoglucomutase |
| EHI_110280 | C4LU80 | LIM zinc finger domain containing protein |
| EHI_110720 | C4LUB9 | START domain-containing protein |
| EHI_114010 | C4M8W7 | Thioredoxin domain-containing protein |
| EHI_117920 | C4LYX6 | Pleckstrin homology (PH) domain containing protein |
| EHI_118000 | C4LYY4 | Uncharacterized protein |
| EHI_118750 | C4LYM3 | Uncharacterized protein |
| EHI_121780 | C4M5C4 | Enhancer binding protein-1 |
| EHI_122310 | C4MBP3 | Thioredoxin domain-containing protein |
| EHI_123390 | B1N5A8 | Thioredoxin domain-containing protein |
| EHI_124300 | C4LZG9 | 60S ribosomal protein L11 |
| EHI_124560 | B1N355 | MHD domain-containing protein |
| EHI_125840 | O15729 | Peptidyl-prolyl cis-trans isomerase |
| EHI_125950 | C4LVY0 | Fe-ADH domain-containing protein |
| EHI_126110 | C4LVZ6 | Ribosomal-S7 domain-containing protein |
| EHI_126920 | C4LWW8 | Asparagine-tRNA ligase |
| EHI_130700 | C4LXE8 | Phosphopyruvate hydratase |
| EHI_131190 | B1N316 | 40S ribosomal protein S16 |
| EHI_134740 | C4M023 | Iron-sulfur flavoprotein |
| EHI_134960 | C4M0Y3 | S-phase kinase-associated protein 1A |
| EHI_136220 | B1N3G7 | RRM domain-containing protein |
| EHI_136380 | Q769I7 | Aminotran-5 domain-containing protein |
| EHI_138380 | C4M3I0 | Alpha-1,4 glucan phosphorylase |
| EHI_140740 | C4M3P6 | Uncharacterized protein |
| EHI_141400 | B1N2R5 | Cysteine protease |
| EHI_141930 | C4LW39 | Protein kinase |
| EHI_148470 | C4LT17 | Aspartate-ammonia ligase |
| EHI_148820 | C4LT49 | 40S ribosomal protein S24 |
| EHI_148850 | B1N2Z3 | 60S acidic ribosomal protein P0 |
| EHI_150390 | C4M220 | Aspartate ammonia-lyase |
| EHI_150490 | C4M230 | Aldehyde-alcohol dehydrogenase |
| EHI_151810 | C4LSM2 | Eukaryotic translation initiation factor 5A |
| EHI_152330 | C4LSS0 | Arginase |
| EHI_152340 | C4LSS1 | Choline/ethanolamine kinase |
| EHI_152650 | B1N2F9 | Flavodoxin-like domain-containing protein |
| EHI_152680 | C4LSV2 | EH-domain containing protein |
| EHI_154450 | B1N5A6 | Uncharacterized protein |
| EHI_155160 | C4LW70 | Serine/threonine-protein kinase TOR |
| EHI_155410 | C4LW92 | Ribosomal-S17-N domain-containing protein |
| EHI_155440 | C4LW95 | Thioredoxin reductase |
| EHI_159160 | C4LX20 | Superoxide dismutase |
| EHI_159480 | C4LX30 | Pore-forming peptide ameobapore A, putative |
| EHI_159620 | C4LX42 | TolA protein |
| EHI_160980 | C4M2R6 | Uncharacterized protein |
| EHI_161070 | C4M2S3 | Uncharacterized protein |
| EHI_161970 | C4M6P0 | Leucyl-tRNA synthetase |
| EHI_165350 | C4M213 | Malate dehydrogenase |
| EHI_166490 | C4M7F5 | Fe-ADH domain-containing protein |
| EHI_166810 | B1N306 | Elongation factor 2 |
| EHI_166850 | C4LY89 | Proteasome endopeptidase complex |
| EHI_166920 | C4LY96 | PPi-type phosphoenolpyruvate carboxykinase 1 |
| EHI_167300 | C4MAF9 | Grainin 1 |
| EHI_167310 | Q9NJW1 | Grainin 2 |
| EHI_168340 | C4M4P4 | ADF-H domain-containing protein |
| EHI_175020 | C4LZM7 | Peptidase-M24 domain-containing protein |
| EHI_175050 | C4LZN0 | Aspartyl-tRNA synthetase |
| EHI_176970 | C4LY22 | Cdc48-like protein, putative |
| EHI_177660 | C4LY19 | Isoleucyl-tRNA synthetase |
| EHI_177990 | C4M553 | Leucine-rich repeat containing protein |
| EHI_178960 | C4LUV9 | Acetyl-CoA synthetase |
| EHI_182670 | C4LV48 | Enhancer binding protein-2 |
| EHI_185280 | C4M3Z6 | Phosphoglucomutase/phosphomannomutase family protein |
| EHI_186480 | C4LVN8 | Eukaryotic translation initiation factor 5A |
| EHI_186820 | C4LVP9 | Protein kinase domain-containing protein |
| EHI_187020 | C4LVR9 | Glyceraldehyde-3-phosphate dehydrogenase |
| EHI_188180 | C4M192 | Phosphoglycerate kinase |
| EHI_188910 | C4M2V8 | Uncharacterized protein |
| EHI_189930 | C4M711 | Uncharacterized protein |
| EHI_192470 | C4M1G7 | Fe-ADH domain-containing protein |
| EHI_195110 | B1N3M4 | S-adenosylmethionine synthase |
| EHI_196940 | C4LVB2 | Heat shock protein 90 |
| EHI_197010 | C4LVB8 | Uncharacterized protein |
| EHI_197340 | C4LVF0 | Sulfotransferase |
| EHI_197480 | C4LVG4 | ADF-H domain-containing protein |
| EHI_198620 | C4LWQ8 | PPi-type phosphoenolpyruvate carboxykinase 3 |
| EHI_198740 | C4LWS0 | 40S ribosomal protein S19 |
| EHI_198750 | C4LWS1 | Polyadenylate-binding protein |
| EHI_198760 | C4LWS2 | Fe-ADH domain-containing protein |
| EHI_198870 | C4LWT3 | Transcription factor BTF3 |
| EHI_199000 | C4LWU6 | Calponin homology domain protein |
| EHI_199050 | C4LWV1 | 2-(3-amino-3-carboxypropyl) histidine synthase subunit 1 |
| EHI_199660 | C4M779 | Uncharacterized protein |
| EHI_200080 | C4M7M1 | Purine nucleoside phosphorylase |
| EHI_200560 | C4M3X2 | J domain-containing protein |
| EHI_202040 | Q9U8X2 | Serine acetyltransferase, putative |

**Supplementary Figure 1.** Gene ontology (GO) enrichment analysis and functional annotation clustering of the 221 secreted proteins immunoprecipitated with the anti-LBPA antibody, were carried out using the GeneOntology database (http://geneontology.org/). GO analysis allows the association of a given gene list with specific functional annotations, which are further divided into functional clusters listed according to an enrichment *P*-value. The 10 most significant enriched GO terms in biological process, molecular function and cellular component branches are presented. All the adjusted statistically significant *P*-values of the terms were negative 10-base log normalized.
